# Related Factors to Streptococcus pneumoniae Invasive Infection and Clinical Manifestations: The Potential Role of Nasopharyngeal Microbiome

**DOI:** 10.3389/fmed.2021.650271

**Published:** 2021-04-20

**Authors:** Beatriz Dietl, Desirée Henares, Lucía Boix-Palop, Carmen Muñoz-Almagro, Javier Garau, Esther Calbo

**Affiliations:** ^1^Infectious Diseases Unit, Hospital Universitari Mútua Terrassa, Terrassa, Spain; ^2^Department of Medicine, Universitat Internacional de Catalunya, Barcelona, Spain; ^3^Institut de Recerca Sant Joan de Déu, Hospital Sant Joan de Déu, Barcelona, Spain; ^4^Center for Epidemiology and Public Health, CIBERESP, Instituto de Salud Carlos III, Madrid, Spain; ^5^Internal Medicine Department, Clínica Rotger, Palma de Mallorca, Spain

**Keywords:** microbiota, nasopharynx, *Streptococcus pneumoniae*, pneumococcal infections, invasive pneumococcal disease

## Abstract

Infections of the lower respiratory tract, such as pneumonia, are one of the leading causes of death worldwide. *Streptococcus pneumoniae* might colonize the upper respiratory tract and is the main aetiological agent of community-acquired pneumonia (CAP). In the last decades, several factors related to the host, the microorganism and the antibiotic therapy have been investigated to identify risk factors associated with the development of invasive pneumococcal disease (IPD). Nevertheless, these factors themselves do not explain the risk of developing disease or its severity. Recently, some studies have focused on the importance of nasopharyngeal (NP) microbiome and its relation to respiratory health. This review presents existing evidence of the potential role of NP microbiome in the development of IPD.

## Introduction

Lower respiratory infections, considering also pneumonia, represent the third cause of death worldwide, with a median of 4.2 million deaths (7.1% of total deaths) per year (1). Combination of pneumonia and Influenza ranked 8th among leading causes of mortality in developed countries. Also, they are the main cause of death due to infection (2). *Streptococcus pneumoniae (S.pneumoniae)* is the most common pathogen implicated in community-acquired pneumonia (CAP) and accounts for two-thirds of the mortality in hospitalized patients (3).

Invasive pneumococcal disease (IPD) refers to an infection confirmed by the isolation of *S.pneumoniae* from a normally sterile site (pneumonia, parapneumonic empyema, meningitis, bacteraemia/sepsis, peritonitis and arthritis) (4). Its incidence, despite its remarkable reduction since the introduction of pneumococcal conjugated vaccine (PCV), is still 10–20/100,000/year in developed countries and could be even higher in developing countries (5–11).

To date, the risk of developing pneumococcal pneumonia and also IPD has been associated with the interplay between host susceptibility and pathogen virulence.

Many factors have been described regarding host susceptibility for the development and prognosis of pneumococcal pneumonia, as age, comorbidities and previous vaccination status (12–15). Severity and mortality in pneumococcal pneumonia are also related to strain characteristics, coinfection and antibiotic treatment (16, 17).

However, these elements do not fully explain by themselves the potential risk for infection and its severity. Despite recent advances in this field, it is still not known why some individuals present asymptomatic NP colonization; others develop localized infection and others, IPD.

The aim of this review was to present the current evidence for the role of different factors –including the potential role of NP microbiome- involved in the dissemination of *S.pneumoniae* from the nasopharynx to other human body sites and its subsequent clinical manifestations.

## Methods

A literature search of PubMed and WOS databases conducted between January 2000 and February 2021 using the parameters [microbiome (MeSH Terms)] OR [infection, streptococcus pneumoniae (MeSH Terms)] OR [pneumonia (all fields)] generated >180,000 abstracts.

Titles and abstracts were assessed, as a rule of thumb, and clinically relevant articles were reviewed. References of the selected articles were also reviewed and those which could fit the topic were read and evaluated as well.

## Host Characteristics

### Baseline Characteristics and Clinical Features

*S.pneumoniae* is a large contributor to mortality worldwide (12, 13, 18). It is the most common documented etiology of severe bacterial respiratory tract infection in children, but also in the adulthood. The spectrum of disease ranges from colonization (especially among children under 5 years of age) to mucosal disease (otitis media, sinusitis, pneumonia), and to invasive infections (bacteremia, meningitis, endocarditis, others) (18).

Increasing age, male gender, toxic habits (as smoking and alcohol abuse), liver or renal disease, solid organ tumors, immunosuppression (HIV infection, asplenia) and higher Charlson Comorbidity Index have been identified as independent risk factors for high mortality among patients with community-acquired, bacteraemic pneumococcal pneumonia.

Among all the host factors previously described, age has been identified as the strongest predictor of death, even in patients without significant comorbidity (13, 19, 20).

### Vaccination

*S.pneumoniae* infection is one of the most vaccine-preventable diseases. In the 1980s and 1990s, the 23-valent-polysaccharide vaccine (PPSV23) was the only direct prevention measure available (12). Polysaccharide antigens produce the activation of mature B cells. However, protein-polysaccharide conjugate pneumococcal vaccines (PCV) have a T-cell-immune response and are effective in immunosuppressed patients (21). In 2000 the 7-valent vaccine (PVC7) -against seven serotypes- was licensed for children in the USA. After that, the increment in relevance of non-vaccine serotypes, conducted to the development of new vaccines: PCV10 (PCV7 plus 1, 5 and 7F), and PCV13 (PCV10 plus 3, 6A and 19A) (22). At present, there are two new conjugate vaccines in development (PCV15 and 20vPnC) (23).

Many investigators have focused on the subsequent impact of young children vaccination in the incidence of IPD in other age groups. Although there are some reports of this incidence being increased (24, 25), there is solid evidence of a sustained decrease in IPD incidence in vaccinated children and adults, -including the immunosuppressed population- supporting the use of PVCs (12, 22, 26–29).

### Genetic Polymorphisms

The innate immune system represents the first non-specific step in host defense. The recognition of pathogens by the host immune system is a necessary requisite for the initiation of a response (15). A bunch of different receptors are placed on the cell surface of the epithelial barrier and on hematopoietic cells. These receptors recognize diverse pathogen antigens. After these pattern recognition, a very complex net of intracellular signaling pathways is triggered in order to develop a response of the host against the pathogen (30).

In the 1980s, genetics was found to be a major determinant of susceptibility to infectious diseases. Extreme-phenotype studies in patients with recurrent IPD were successful in the identification of factors associated with increased susceptibility (31). Different genetic variants in the proteins involved in the signaling pathways have been identified in the last 20 years as elements that either increase the risk or confer protection against pneumococcal pneumonia. A recent meta-analysis showed that variants in *CD14* and *MBL2* genes were associated with susceptibility to pneumococcal disease. Several other host genetic polymorphisms have been identified that potentially influence susceptibility and outcome of pneumococcal disease, although most of these findings have not been confirmed in independent studies (32).

Thus, all these findings support the hypothesis that genetic variants may explain, at least partially, the host susceptibility to pneumococcal pneumonia and IPD.

## Bacterial and Treatment Factors

The outcome of IPD can be affected by host factors, such as age (the very young and the very old), underlying conditions, low socioeconomic status and quality of life; but also by bacterial factors such as the serotype (16).

### Serotype, Invasiveness, and Severity

At least 100 serotypes of *S.pneumoniae* has been identified, based on antigenic differences in their capsular polysaccharides (18).

Main serotypes found in NP carriage and invasive disease are quite similar worldwide (33, 34). Likewise, the frequency by which a certain serotype causes invasive disease per carriage episode is a stable property along the time. There is an inverse relationship between frequency of carriage and invasiveness: serotypes which are less commonly carried cause more frequently invasive disease whilst the serotypes most prevalent in carriage are less invasive (35). Also, it has been described that serotypes which have less risk of causing invasive disease are associated with more severe disease and mortality, even in healthy individuals (36–38).

According to the studies of Brueggemann et al. serotypes 1, 3, 4, 5, 7F, 8, 9A, 9V, 12F, 14, 18C, and 19A are considered highly-invasive serotypes while the remaining serotypes were considered non-highly-invasive or opportunistic (39).

A meta-analysis of IPD outcome by serotype in 2011 was consistent with these findings: there is a relationship between IPD and pneumococcal serotype. The risk for IPD has a direct relation with the serotype prevalence, and is inversely correlated with invasiveness. Besides, the findings of this study suggested a potential mechanism for the epidemiologic relationships between serotypes. The most prevalent among carriage isolates are the more heavily encapsulated, which rarely cause bacteraemic invasive disease, but can cause more severe disease when they do invade (16).

### Impact of Initial Antimicrobial Treatment

The effect of *S.pneumoniae* resistance to antimicrobials and subsequent discordant antimicrobial therapy (DAT) has been largely investigated with controversial results. While some studies observed that patients who received DAT did not have a higher mortality rate (40), others concluded that discordant antibiotic prescribed at admission was strongly related with higher mortality (41).

More recently, in a study conducted in Spain to assess the relationship between empirical antibiotic treatment and mortality in CAP due to *S.pneumoniae*, the authors evaluated some clinically relevant situations. Among the subgroup of bacteraemic patients, the choice of empirical antibiotic combination other than monotherapy with β-lactam or macrolide combination of β-lactam and macrolide or levofloxacin alone or in combination was associated with higher mortality (17).

Regarding beta-lactam therapy, pharmacokinetic and pharmacodynamic studies suggest that there is no association between mortality and penicillin MIC ≥2 mg/mL in patients that receive penicillin monotherapy at a dosis of ≥10 MU per day (usually ≥12 MU per day) (42).

## Nasopharyngeal Microbiome and Respiratory Disease

The human body is considered to be a super-organism which is composed of more microbial cells than body cells (43). The term *microbiome* was suggested by Joshua Lederberg and refers to the collective genomes of our endogenous microbes or microbiota. This human microbiota plays essential roles in human body functions, as the maintenance of the integrity of the epithelium (44), the modulation of the immune response (45, 46), and the “colonization resistance” to avoid the invasion of pathobionts, microorganisms which can live as commensal symbionts but, under not yet completely characterized circumstances (related to host genetics, microbial context, etc.) could act as pathogens and cause invasive disease (47–52). Research on this topic has largely focused on the gut microbiota; however, recent studies provide growing evidence of the importance of respiratory ecosystems on human health.

Classically, the lung has been considered sterile. More recently, the use of molecular typing [especially quantitative PCR for 16S ribosomal RNA (rRNA)] has allowed identification of pathobionts in culture-negative respiratory specimens (53, 54). These techniques have permitted the identification of five phyla in the lungs of healthy individuals: Actinobacteria, Proteobacteria, Bacteroidetes and Firmicutes (55), including low levels of oral bacteria, like those of the genera *Prevotella* and *Veionella* (56, 57). The composition of the lower respiratory tract (LRT) microbiota seems to depend on the upper respiratory tract (URT) microbiota composition, due to aspiration of oropharyngeal secretions, micro aspiration (i.e., while sleeping) or direct contact by continuous mucosa (48, 58, 59). Some studies report that there are indistinguishable microbiota patterns along the respiratory tract in healthy individuals, with decreasing biomass content from upper to lower respiratory tract (56, 60). One study found high viral and bacterial microbiota concordance between nasopharyngeal and endotracheal samples during the course of childhood LRTIs, suggesting that upper respiratory tract (URT) samples could be a valid proxy for lung microbiota during disease states (61). These findings have now changed the assumption that the lower airways are normally sterile (62).

Lung colonization by microbiota starts after birth and therefore remains unaltered during the whole lifetime (63–65). Gut and lung microbiota are actually connected in the gut-lung immunity axis. The metabolites produced by the gut microbiome (short-chain fatty acids) can reach other organs and have some influence in the respiratory diseases (66, 67). Recently, it has been shown the role of the gut-lung immunity axis in the pneumococcal pneumonia, by whom the integrity of the gut microbiota plays a main role in the host defense (68).

The colonization of the URT is the essential first step for a respiratory infection, either of the URT, LRT or a disseminated infection (33). Between the different species of the microbiota, there can exist positive interactions (as mutualism or commensalism) or negative interactions (antagonism) (48). Thus, some members of the microbiome have potential advantageous effects on ecosystem equilibrium, health and functionality (69). Examples of these members in the URT microbiota are *Dolosigranulum* spp. and *Corynebacterium* spp., as they have been related to respiratory health and negatively associated to disease caused by pathobionts, such as *S.pneumoniae* (48, 70, 71). The impediment for the pathogens to find necessary resources because the available nutrients might be used by a diverse local microbiome is one of the mechanisms described behind colonization resistance (48). Certain microorganisms have clearly been identified as having the ability to exclude pathogens from the NP ecosystem (72). In the case of *S.pneumoniae*, for instance, the release of free fatty acids -which come from the skin surface tiacylglycerols- by the *Corynebacterium* species *C.accolens*, has been described as one of the mechanisms which could inhibit pneumococcal growth (71). Furthermore, colonization resistance may be enhanced by interactions with the host immune system (48).

The URT is considered to be a major reservoir for potential pathogens, including *S. pneumoniae*, from where it could expand and arrive to the lungs and potentially cause infection (33). Thus, the study of the microbiome in the respiratory tract will shed light in the understanding of susceptibility to pulmonary infections, pneumococcal pneumonia or even IPD.

Main advances on this field have been achieved on pediatric population and adult population with chronic respiratory diseases, as chronic obstructive pulmonary disease (COPD). A relation of recent studies addressing the relationship between respiratory microbiome and infection is showed in Table 1. In samples from the respiratory tract of children, it has been described a high inter-individual variability in bacterial composition (81). Despite this variability, the bacterial composition has been proposed to be within discrete categories where some bacterial taxa dominate the community. In infants between 1 and 3 months of age, the hypopharynx was found to have five “pneumotypes” which follow concrete trajectories during the child's development (82). Distinct microbiota profiles have been detected in young children (under 2 years old). Early-life profiles are associated with microbiota stability and change patterns of change during the two first years of life. During the first 2 years, stable microbiota profiles were characterized by an early colonization of *Moraxella*, and *Dolosigranulum* combined with *Corynebacterium*, whereas instability was associated with profiles dominated by *Haemophilus* and *Streptococcus*. Moreover, infant feeding and frequency of respiratory infections in the parents were associated with the patterns of change. A history of breastfeeding and a reduced number of consecutive respiratory infections were associated with stable microbiota profiles (63).

**Table 1:** Studies of respiratory microbiota related to lower respiratory tract infections.

**Reference**	**Population, number of patients**	**Methods**	**Main findings**
Zhou et al. (73)	Adults, 101	Observational, prospective	•*Streptococcus, Staphylococcus, Pseudomonas*, and *Acinetobacter* were identified as the most important species in hospital-acquired pneumonia
Bousbia et al. (74)	Adults, 210	Observational, prospective, case-control	•*Pseudomonas aeruginosa* and *Streptococcus* sp. were as common in pneumonia patients as in controls •Different microbiota patterns were presented in different forms of pneumonia
Iwai et al. (75)	Adults (HIV-infected), 15	Observational, prospective, case-control	•HIV-patients had increased abundance of species including several pathogenic microorganisms compared to controls
Chaban et al. (76)	Adults, 65	Observational, cross-section	•Respiratory microbiota composition of H1N1 patients relied on 11 different patterns dominated by one or two microorganisms
Leung et al. (77)	Adults, 22	Observational, prospective, longitudinal	•H1N1 infection could provide with previously unrecognized pathogens which could travel to the LRT and cause infection in weakened patients
Biesbroek et al. (63)	Children, 60	Observational, prospective, longitudinal	•Distinct patterns of NP microbiota in healthy children from infancy were associated with stable composition and less susceptibility for further respiratory infections
de Steenhuijsen Piters et al. (78)	Adults (elderly), 191	Observational, prospective, longitudinal with two cohorts	•Pneumonia in either young and elder adults is related with URT microbiome dysbiosis •Dysbiosis was characterized by bacterial overgrowth of *S.pneumoniae, Rothia* ad *Lactobacillus* and absence of anaerobic bacteria
Man et al. (61)	Children, 29	Matched case-control study	•Presence and severity of LRTI was associated with the URT microbiota composition •*S.pneumoniae* and *Haemophilus* were associated with disease
Camelo-Castillo et al. (70)	Children, 56	Observational, prospective, longitudinal	•Three nasopharyngeal microbiota patterns significantly associated with case and healthy controls •The dominated by *Streptococcus* sp. type was more frequent in cases; the type mainly composed by *Dolosigranulum* sp. was more frequent in controls.
Chapman et al. (79)	Children, 358	Retrospective	•NP colonization of *Moraxella* at early infancy was associated with less microbiota diversity and co-colonization with *S.pneumoniae* and *H.influenzae* •Colonization with *Moraxella* supposed an increased risk for respiratory illnesses
Rueca et al.(80)	Adults, 31	Observational, cross-section, case-control	•Patients with SARS-CoV-2 infection had a complete depletion of *Bifidobacterium* and *Clostridium* •In the case group, *Salmonella, Scardovia, Serratia* and *Pectobacteriaceae* were present

*Abbreviations: LRT: lower respiratory tract, NP: nasopharyngeal*.

*URT: upper respiratory tract, LRTI: lower respiratory tract infections*.

Studies of the early URT microbiota in children have connected the early microbiota to the development of disease later in life and to the impact of important drivers. Accelerated microbiota maturation (enriched of *Neisseria* spp. and (facultative) anaerobes, mainly oral species including *Prevotella*) is associated with microbiota instability and increasing number of respiratory tract infections (RTIs) over the first year of life. These changed dynamics could be observed within the first month of age and, as stated previously, might be connected to important drivers: a healthy microbiota development and stability might be linked to vaginal delivery and breastfeeding, although this connection is controversial (83, 84).

In 2020 Chapman et al. conducted a large, retrospective study in children in order to address the reasons why some infants are more susceptible to respiratory infections than others (79). In this study, the cohort was divided into two groups: “infections and allergy prone group (IAP)” and “non-infection and allergy prone group (NIAP).” Males and daycare attendance were independently identified as risk factors for IAP. In terms of microbiome characteristics, colonization of NP in an early-age (between 6 and 36 months) with any of the three pathobionts most frequently associated with respiratory infections (*S.pneumoniae, H.influenzae* and *M.catarrhalis*) was associated with the IAP group.

More concretely, regarding the potential relationship between pneumococcal infection and microbiome, a case-control study was conducted in Spain to compare the nasopharyngeal microbiota of children with IPD (cases) and healthy children (controls) -representative of a healthy nasopharyngeal niche. In this study, bacterial richness and diversity were significantly higher in the groups of children who developed IPD. Three clusters corresponding to three different nasopharyngeal-types (nasopharyngeal types A, B or C, respectively) were observed. These patterns were significantly associated with the classification of the patients into cases and controls. The most frequently detected pattern in children with IPD (observed in 50.0% of the cases) was nasopharyngeal-type B, mostly represented by the genera *Streptococcus* (36.9%)*, Staphylococcus* (21.3%), *Veillonella* (9.8%) along with a diversity of anaerobic genera (*Prevotella* and *Porphyromonas*). The other two nasopharyngeal types [type C, composed of *Haemophilus* (52.1%), *Moraxella* (31.4%) and *Streptococcus* (11.4%)] and type A [composed mainly of the genera *Dolosigranulum* (44.3%), *Moraxella* (29.3%) and *Haemophilus* (10.5%)] were detected in 32.1 and 17.9% of children with IPD, respectively. Conversely, the nasopharyngeal-type A was the most frequently related with healthy controls, leading to the hypothesis—and according to previous studies (63)—that *Dolosigranulum* sp. could confer resistance against pneumococcal infection (70). A clear imbalance was observed with a high frequency of *Veillonella* and other oral microorganisms which were be relatively infrequent in controls. These results were surprising given that higher bacterial diversity has been associated with health, and lower bacterial diversity has been associated with disease (85).

In adults, a recent study was designed to evaluate the characteristics of the NP microbiota in the pneumococcal acquisition (86). At baseline, samples from the NP of the healthy volunteers enrolled, showed mainly species from four genera: *Corynebacterium, Dolosigranulum, Staphylococcus* and *Streptococcus*. From these frequently found bacteria, the NP microbiome could be divided into five different profiles (A-E). Profiles B-E were dominated by *Staphylococcus* sp., *Streptococcus* and *Corynebacterium* sp. or *Corynebacterium* sp. and *Dolosigranulum* sp. combined, whilst profile A showed more diversity. The latter profile was more frequently associated with *S.pneumoniae* carriage (86). In other study, where bronchoalveolar lavage samples were analyzed, two pneumotypes in asymptomatic adults were found: one of them, enriched with supraglottic-characteristic bacteria *Prevotella* and *Veillonella* was associated with higher levels of inflammatory markers (57).

A large revision on the role of microbiome in the innate immune response related to chronic lung diseases (CLD) was published in 2020 (87). COPD is a progressive inflammatory disorder characterized by persistent airflow limitation, obstructive bronchiolitis and parenchymal destruction (88) with a high disease burden and related mortality rates. An specific pattern of microbiota has been identified in COPD patients, in comparison with healthy controls (87). *Proteobacteria* were more frequent in COPD than *Bacteroidetes* (and *Prevotella* spp. was specially reduced) (89). COPD mortality is higher during periods of acute exacerbations (AECOPD). During AECOPD, patients show a temporally dynamic sputum microbiome (90). In addition, the sputum microbiome profile at first day of exacerbation is related to 1-year mortality. The absence of *Veillonella* increases mortality risk by 13-fold whereas the presence of *Staphylococcus* increases the risk by 7-fold. When these two factors were combined in the same individual, 1-year mortality risk in COPD increased by 85-fold (91). Thus, reduced diversity of microbiome in sputum of AECOPD patients confers a poor prognosis, which is consistent with the previous association of higher bacterial diversity and health (90, 91).

Asthma has largely been studied on its relationship with microbiome. Several studies have identified, on one hand, the respiratory microbiota pattern related to asthma (high abundance of *Haemophilus influenzae, Streptococcus pneumoniae, Staphylococcus aureus*, and *Moraxella catarrhalis*, which can potentially play a pathogenic role) (87, 92) and on the other, that these species have a role on the inflammatory response which could determine the outcomes in asthma (93).

Apart from CLD, a limited number of studies have investigated the possible influence of the URT microbiome on the development of LRTIs in adult patients. In general, published studies t include moderate sample sizes without a healthy control group (73, 74), or specific risk groups like HIV (human immunodeficiency virus)-infected patients (75), or patients infected with the pandemic H1N1 influenza virus (76, 77).

In the last year, the spreading of SARS-CoV-2 a novel *beta-coronavirus*, has provoked a world pandemic. COVID-19, the disease caused by this new virus, ranges from an asymptomatic estate to a severe pneumonia associated to a potentially lethal adult respiratory distress syndrome (SDRA) (94). In the search of answer to the question of why this respiratory virus could affect humans in such a different way, the role of respiratory microbiota has been put on the spot. A very recent review about the role of respiratory microbiota in COVID-19 patients (95) concluded, based on previous evidence, that the dysbiosis in the microbiota in COVID-19 patients would potentially lead to infection or progression of the disease. However, studies on the characteristics of microbiome in COVID-19 patients are still scarce and conducted in a small number of patients to make them suitable for extrapolating conclusions (80, 96).

Although it seems reasonable that the respiratory microbiome composition may play a role in the development of IPD, there is, to our knowledge, one single study that has investigated this association in elderly and young adults (78). In this study by de Steenhuijsen et al. the differences in microbiota profiles between patients with pneumonia and their healthy controls were identified as an independent factor. In the elderly, pneumonia was associated with *Rothia, Lactobacillus* and *Streptococcus (pseudo) pneumoniae* whilst healthy adults showed greater diversity and higher richness of especially three different patterns of microbiome (*Prevotella melaninogenica, Veillonella* and *Leptotrichia*) in the oropharynx samples (78).

## Therapeutic Options in Respiratory Infections

One of the main applications of the knowledge of microbiota composition are the potential therapeutic options. The modification of microbiota profiles to a protective pattern which could lead to a less degree of tissue inflammation, damage and therefore disease, is an attractive approach for this novel research (97).

A previous systematic review evaluated 23 trials in children to evaluate the efficacy of probiotics for prevention and treatment of recurrent respiratory infections (RRI) (98). After meta-analysis and considering the available evidence, probiotics were postulated as a possible alternative therapeutic option for RRI in children. However, the probiotic strain, dosage and administration forms were very heterogeneous among the different studies analyzed.

Human-associated microorganisms are able to produce secondary or specialized metabolites (natural products) which could mediate in the interactions between host and microbes and between microbes themselves (99). These natural products released by non-pathogenic species could be a novel source of antimicrobials, due to their antimicrobial activity against pathogen species (99, 100). More recently, Manti et al. conducted a prospective study in order to prove the *a priori* protective role of *Streptococcus oralis* and *Streptococcus salivarius* using them as a therapeutic option (101). Ninety-one children between 1 and 12 years old were prospectively included in a single-open study, in which a nasal spray composed of *Streptococcus oralis* 89a and *Streptococcus salivarius* 24SMBc was administered. After probiotic treatment, clinical improvement was reported, even in children with previous history of atopy or allergies. However, results were only applicable for the initial 3-months, due to a lack of follow-up period in the study protocol.

Phage therapy has arisen as a promising therapeutic tool in the era of antimicrobial resistance (102). Phage lysins, which are encoded by phages, are cell wall hydrolases that selectively act against different peptidoglycan bonds. These proteins can attack especially Gram-positive bacteria by splitting the bacterial cell wall (103). Some lysins have demonstrated activity against *S.pneumoniae* (104). In a recent research, a new phage lysine, 23TH_48 has been postulated as a potential therapeutic weapon for pneumococcal infections, combined with other lysins or antibiotics. This sinergyc combinations could be used to broaden the spectrum of action and improving their antimicrobial activity (103).

## Discussion

Recent advances in molecular typing techniques have identified not only that the lungs are not sterile –as was classically believed- but also that the respiratory tract is colonized by microbial species, which change between healthy individuals and those affected with respiratory diseases (53–57). Microbial populations play an important role in health. Along the human airways, structures above the vocal cords are exposed to high bacterial burden producing contamination of lower airway secretions from the URT (57).

We have reviewed the most important factors known to be associated with pneumococcal disease and we have focused on the available evidence of the role of respiratory microbiome in the development of respiratory infections in children and adults. It has not been established how these previously identified factors might impact in respiratory microbiota development and thereby in susceptibility to LRTIs. Despite the many comorbidities and conditions that have been identified as risk factors for the development of IPD, only the extremes of age (<5 years and >60 years old) have been strongly consistently found to be a major risk for IPD (13, 19, 20). Lung colonization is believed to start early after birth, with different profiles related with different factors, such as infant feeding. Stable patterns were associated with less risk of respiratory infections whilst changing patterns were associated with increased incidence of respiratory infections (63). The presence of these changing microbiota patterns–with *Haemophilus* and *Streptococcus* dominant profiles- in children under 2 years of age could explain the higher incidence of IPD in this population. On the other hand, changes on respiratory microbiota through age in the adults were also associated with a higher risk for LRTI. The absence of anaerobic species in the very old –a phenomenon linked to increasing age- could be associated with a high susceptibility for pneumonia at the extreme of life (78).

Vaccination against pneumococcal has changed the epidemiology of the pneumococcal infections and, despite controversial results of previous studies, there has been a decrease in IPD incidence in the vaccinated population, even in the immunosuppressed (12, 22, 26–29). Pneumococcal vaccination has had also an impact in NP microbiota characteristics. In spite of a lack of evidence for a different composition between vaccinated and non-vaccinated children, a higher abundance was identified in patients after PCV 10 vaccine. This complexity could explain that, after vaccination, individuals are less prone to suffer acute respiratory tract infections (105).

Several host genetic polymorphisms which control the pathways of the immune system to combat bacterial infection have been identified as a risk factors for protection or susceptibility to pneumococcal pneumonia (32). Considering that some factors -as, for example, CXCL16- are regulated by microbiota through modulation of the quantity of iNKT cells in the gut and lung, leading to a higher tissue inflammatory response (46), these genetic variations could lead to a different expression of signaling proteins which could be, in turn, modified by the different microbiota patterns conferring more risk or protection against pneumococcal pneumonia.

Studies in recent years have focused on characterization of the respiratory microbiota, and concluded that the LRT microbiota composition comes from the URT (48, 53–59). Due to its role in the regulation of the immune response and inflammation (45, 46), the respiratory microbiome has been associated to the development and exacerbations of chronic lung diseases, as asthma and COPD (87–93). However, the most relevant findings in this review have been the implication of respiratory microbiome in pneumonia. Thus, colonization of the NP with either *S.pneumoniae, H.influenzae* or *M.catarrhalis* in children was associated with a tendency for respiratory infections and allergy in the pediatric period. Moreover and related to the scope of this review, in the study conducted by Camelo-Castillo et al. different microbiota patterns on the NP of children, were associated to IPD or asymptomatic colonization in this population (70).

Conversely, in the adult less is known on the impact of microbiome development of LRT infections, including pneumonia and more specifically, pneumococcal pneumonia and IPD. De Sreenhuijsen et al. found -in contrast with the previous studies in children- that anaerobic species were highly represented in old patients with pneumonia, who also had a less diverse and rich oropharyngeal microbiota profile (78).

The identification of microbiota profiles associated to IPD or asymptomatic colonization may be of clinical value as disease biomarkers. According to recent encouraging data about the potential role of probiotics in the treatment and prevention of respiratory infections (mainly in pediatric population) (97, 98, 101), the characterization of beneficial bacteria in adults -preventing or protecting against pneumococcal infection- would allow integrating those microorganisms in a probiotic preparation for the treatment or prophylaxis of pneumococcal infections and IPD.

The main limitation of our study is that we have tried to conduct an unbiased, accurate review of the most relevant literature regarding the evidence on the role of the respiratory microbiota in the development of pneumococcal pneumonia and especially, IPD. However, some relevant publications could have gone unnoticed by our research system and therefore not included in the current literature review.

Finally, considering that the microbial ecosystem of adults is relatively stable in the absence of gross perturbations, the role of microbiome in IPD in children might be likely reproducible in adults.

Data regarding the potential relationship between NP microbiome and the risk of developing IPD in adults, viral coinfection and severity of disease are scarce, and specific research in this area is needed. Although NP microbiome in patients with IPD has not been properly characterized yet, there seem to be discordant results between pediatric and adult populations. New, longitudinal studies, with larger number of participants and a homogeneous system to collect samples should help to elucidate the potential role of the previously observed microbial species in adults and their relationship with increasing or reducing risk for the development of respiratory infections, especially IPD.

## Data Availability Statement

The original contributions generated for the study are included in the article/supplementary material, further inquiries can be directed to the corresponding author/s.

## Author Contributions

DH, EC, LB-P, and CM-A contributed to the conception and the design of the review. DH, EC, LB-P, and BD contributed to the literature research and selection. JG contributed to the coordination of the review. BD and EC were the major contributors in writing the manuscript. All the authors read and approved the final manuscript.

## Conflict of Interest

The authors declare that the research was conducted in the absence of any commercial or financial relationships that could be construed as a potential conflict of interest.
